# Cranial morphological variation of *Ctenomys lami* (Rodentia: Ctenomyidae) in a restricted geographical distribution

**DOI:** 10.1590/1678-4685-GMB-2023-0130

**Published:** 2023-11-13

**Authors:** Rodrigo Fornel, Renan Maestri, Pedro Cordeiro-Estrela, Daniela Sanfelice, Thales Renato O. de Freitas

**Affiliations:** 1 Universidade Regional Integrada do Alto Uruguai e das Missões, Campus Erechim, Departamento de Ciências Biológicas, Erechim, RS, Brazil.; 2 Universidade Federal do Rio Grande do Sul, Departamento de Ecologia, Porto Alegre, RS, Brazil.; 3 Universidade Federal da Paraíba, Departamento de Sistemática e Ecologia, João Pessoa, PB, Brazil.; 4 Instituto Federal de Educação, Ciência e Tecnologia do Rio Grande do Sul, Campus Restinga, Porto Alegre, RS, Brazil.; 5 Universidade Federal do Rio Grande do Sul, Departamento de Genética, Porto Alegre, RS, Brazil.

**Keywords:** Cranial shape, geometric morphometrics, chromosomal polymorphism, chromosome rearrangements

## Abstract

The relationship between chromosomal and morphological variation in mammals is poorly understood. We analyzed the cranial size and shape variation in *Ctenomys lami* concerning to the geographic variation in their chromosome numbers. This subterranean rodent occurs in a narrow range of sand-dunes in the Coastal Plain of southern Brazil. This species presents a high karyotypic variation with diploid numbers varying from 2n = 54 to 2n = 58, involving the fission and fusion of chromosome pairs 1 and 2. Due to different chromosome rearrangement frequencies along their geographic distribution, four karyotypic blocks were proposed. This study, explored cranium shape and size variation in geographical, chromosomal polymorphism, and chromosome rearrangements contexts to test whether the four karyotypic blocks reflect morphologically distinct units. For this, we measured 89 craniums using geometric morphometrics and used uni and multivariate statistics to discriminate the predicted groups and test for an association among chromosomal and morphological variation. Our results show the size and shape of sexual dimorphism, with males larger than females, and support the existence of four karyotypic blocks for *Ctenomys lami* based on morphological variation. However, our results do not support a direct relationship between chromosomal and cranial morphological variation in *C. lami*.

## Introduction

Chromosomal polymorphism has played an important role in speciation, basically by forming efficient barriers to gene flow and, consequently, leading to the differentiation process (Faria and Navarro, 2010; Dobigny et al., 2017; Galindo et al., 2021). The genus *Ctenomys* presents a high diversity in diploid number, in levels intra and interspecific, ranging from 2n = 10 for *C. steinbachi*, to 2n = 70 for *C. pearsoni* (Reig et al., 1990; Ortells and Barrantes, 1994; Freitas, 2016). However, whether intraspecific chromosomal polymorphism is correlated with morphological variation has yet to be understood.

The genus *Ctenomys*, a highly diverse genus of subterranean rodents popularly known as tuco-tucos (Freitas et al., 2021). Their about 70 species (Bidau, 2015; Teta and D’Elía, 2020; D’Elía *et al*., 2021; De Santi et al., 2021; Mapelli et al., 2022; Verzi et al., 2023) have a Pliocene origin, and virtually all diversification events took place during Quaternary (Verzi *et al*., 2021; De Santi *et al*., 2021), making tuco-tucos one of the most explosive mammalian radiations. This rapid diversification within different South American biomes has been inputted to the chromosomal mode of speciation (Freitas *et al*., 2021) because of their considerable karyotypic diversity, both inter and intraspecifically with diploid numbers ranging from 2n = 10 to 2n = 70 (Reig and Kiblisky, 1969; Kiblisky *et al*., 1977; Freitas and Lessa, 1984; Reig *et al*., 1990; Massarini et al., 1991; Freitas, 1997, 2001, 2021; Slamovits et al., 2001; Freitas *et al*., 2021). In fact, their patchy distribution and solitary mode of life (except for *C. sociabilis*) would favor the fixation of chromosomal rearrangements acting as post-zygotic barriers to gene flow. The genus is considered one of the most speciose mammalian genera, with probably the highest rate of chromosomal evolution among mammals (Cook and Lessa, 1998; Lessa and Cook, 1998; Mascheretti et al., 2000; Freitas, 2021).

**Figure 1 -:**
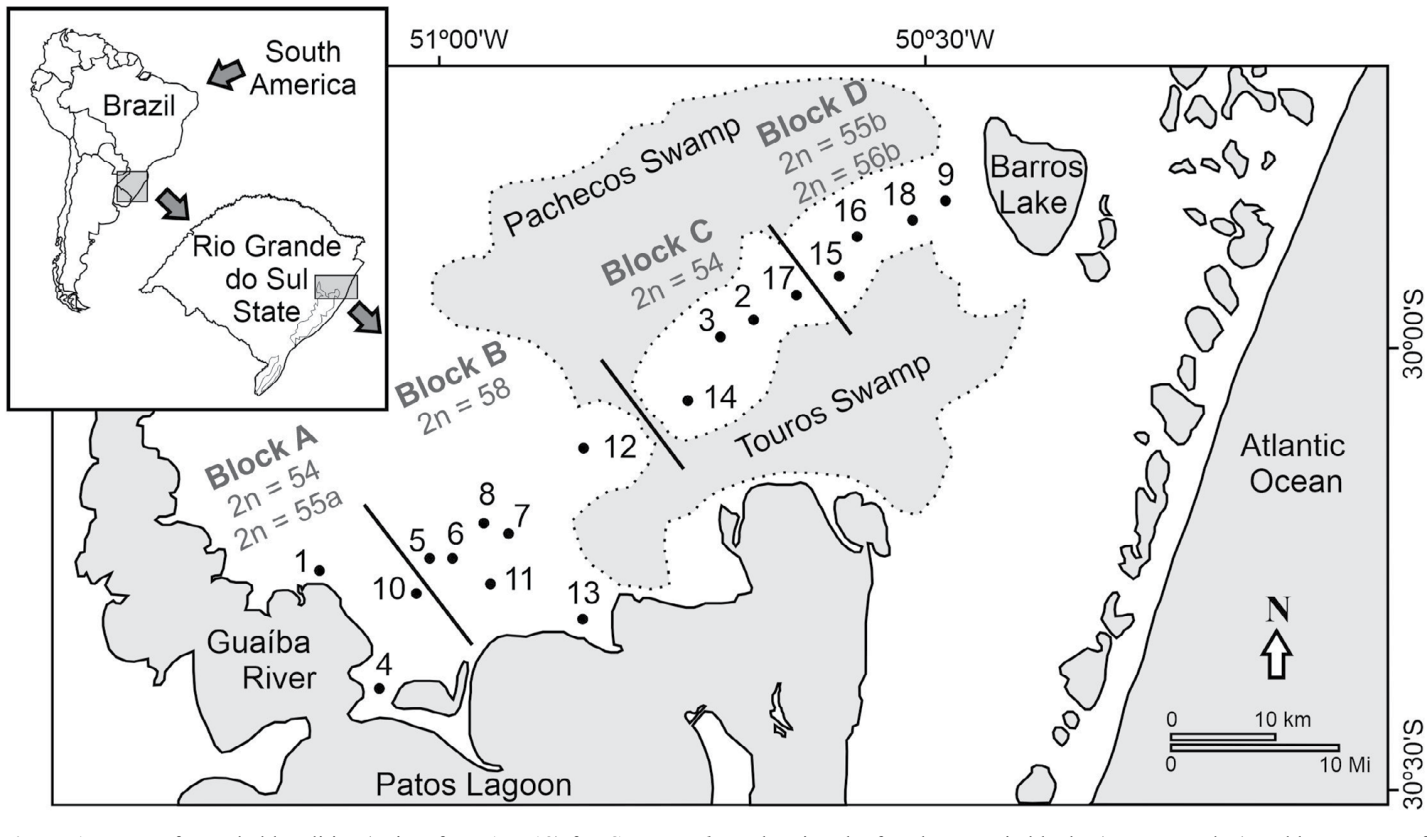
Map of sampled localities (points from 1 to 18) for *Ctenomys lami* showing the four karyotypic blocks (A, B, C and D) and karyotypes of specimens used in this study. The references for each localities number are listed in File S1.

In *C. lami*, those above characteristics of tuco-tucos have evolved to an extreme since it presents the greatest chromosomal variability within the smallest geographic distribution, approximately 940 km^2^ (Freitas, 2001; El Jundi and Freitas, 2004; Freitas, 2007). *C. lami* is found endemically in a region of sandy soils of surrounded by marshes and lakes, known as the Coxilha das Lombas (El Jundi and Freitas, 2004), in the Coastal Plain of State of Rio Grande do Sul in southern Brazil (Figure 1). Added to urbanization and human occupation, turn this species into a target for conservation (Fernandes et al., 2007; Lopes and Freitas, 2012). Seven different diploid numbers have been described for this species, 2n = 54, 55a, 55b, 56a, 56b, 57 and 58, involving the fission/fusion of the chromosomes 1 and/or 2 thus having a wide variation in the fundamental number of autosomal arms (FNa) ranging from 74 to 84 (Freitas, 1995, 2001, 2006, 2007). 

As expected by the model of chromosomal speciation, karyotypes are not randomly distributed. Freitas (1990, 2007) divided the species into four karyotypic blocks based on the spatial distribution of its karyotypes in the Coxilha das Lombas: block A with 2n = 54, 55a and 56a; block B with 2n = 57 and 58; block C with 2n = 54 and 55a; block D with 2n = 55b and 56b (Figure 1). However, Moreira et al. (1991), using protein polymorphisms in *C. lami*, did not find four karyotypic blocks but showed two major groups. Where them found karyotypic blocks A and B from the first major group and the second major group by blocks C and D, with allele frequencies in a clinal pattern of geographical variation (Moreira *et al*., 1991). These two major groups are separated by a natural barrier, the connection of two swamps in the middle of the distribution of *C. lami* (Figure 1). El Jundi (2003) analyzed microsatellite loci variation within this species and found allelic homogeneity among karyotypic blocks suggesting the presence of gene flow between blocks. Lopes and Freitas (2012) assessed the genetic geographical structure of *C. lami* using mitochondrial DNA control region, cytochrome c oxidase subunit I sequence, and microsatellite loci. In a stepping-stone model, they observed an isolation-by-distance pattern with a clinal genetic variation that was not associated with different karyotypes (Lopes and Freitas, 2012).

Chromosomal rearrangements, such as fissions, fusions, duplications and deletions, can alter the expression levels of certain genes, leading to phenotypic alterations (Muñoz-Muñoz et al., 2011). These can be changes in morphology, physiology and even behavior even leading to speciation (Patton and Sherwood, 1983; Muñoz-Muñoz *et al*., 2011). In the genus *Ctenomys*, efforts have already been made to better understand the role of chromosomal evolution in morphological variation, especially in the cranium and mandible (Freitas, 1990; Fernandes et al., 2009; Fornel et al., 2010, 2018). However, in a species vith such a restricted distribution and with such high chromosomal variation, such as *C. lami*, these relationship between chromosomal and morphological variation have not yet been fully clarified. 

Here we explore the variation in the cranium shape of *C. lami* in (1) geographical context, testing space distances among populations and the four proposed karyotypic blocks, (2) in terms of chromosome number (diploid number), and (3) in terms of chromosome rearrangements. We hypothesized was that the cranium variation of *C. lami* would follow a geographic pattern correlated with the chromosomal blocks, resulting in morphological cline coinciding with the chromosomal rearrangements. 

## Material and Methods

### Sample collection

We examined 89 specimens of *C. lami* from four karyotypic blocks, the same proposed by Freitas (1990, 2007), blocks A (n = 25), B (n = 17), C (n = 18), and D (n = 29), or by cranium specimens by karyotypes, 2n = 54 (A) (n = 19), 2n = 54 (C) (n = 18), 2n = 55a (n = 6), 2n = 55b (n = 5), 2n = 56b (n = 24), and 2n = 58 (n = 17). Unfortunately, in the collection there were no crania of individuals 2n = 56a and 2n = 57. All of them were adults, according to by El Jundi and Freitas (2004) definitions. The sampled specimens used in this study are deposited in the Coleção de Mamíferos of the Departamento de Genética, Instituto de Biociências, Universidade Federal do Rio Grande do Sul, Porto Alegre, Brazil. The sex and karyotype of the individuals used in this study were known and were collected at the sites shown in Figure 1. The list of the specimens examined is given in Table S1 and File S1. 

### Geometric morphometrics

**Figure 2 -:**
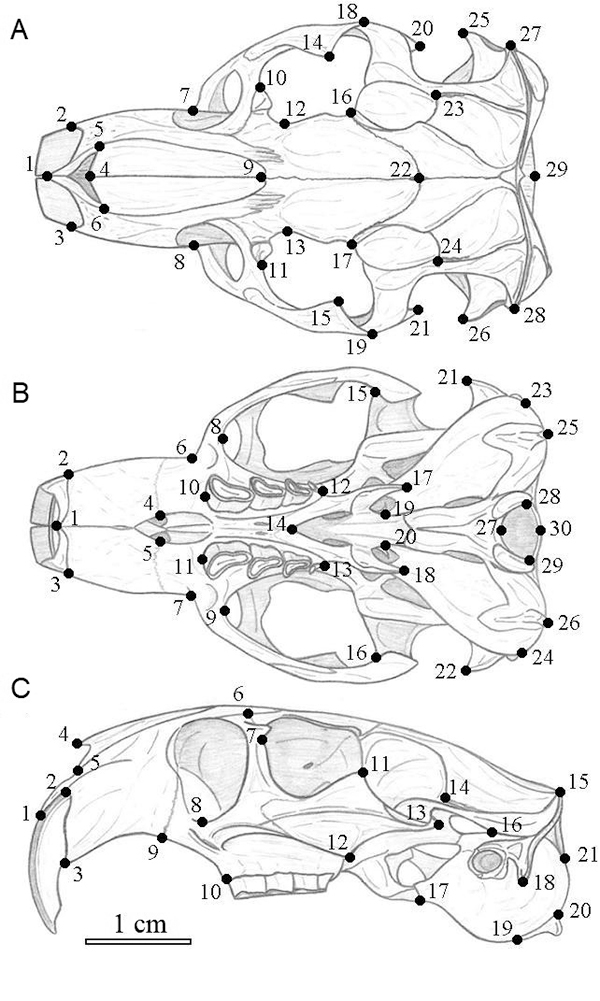
Cranium of *Ctenomys lami* with the location of morphological landmarks for dorsal (A), ventral (B), and lateral (C) views of the cranium (adapted from Fornel et al., 2010, 2018). See File S2 for anatomical description of each landmark.

Each cranium was photographed on the dorsal, ventral, and lateral views with a digital camera of 3.1 Megapixels of resolution (2048 × 1536). We used the same landmarks proposed by Fernandes et al. (2009) for cogeneric species *C. torquatus* and *C. pearsoni*, defined 29, 30, and 21 two-dimensional morphological landmarks for dorsal, ventral, and lateral views of the cranium, respectively (see Figure 2 for landmark location and File S2 for description). The coordinates of each landmark were obtained using tpsDig 1.40 software (Rohlf, 2004). Coordinates were superimposed using a generalized Procrustes analysis (GPA) algorithm (Dryden and Mardia, 1998). GPA removes differences unrelated to the shape, such as scale, position, and orientation (Rohlf and Slice, 1990; Rohlf and Marcus, 1993; Bookstein, 1996a,b; Adams et al., 2004). We symmetrized both sides of the cranium from landmarks of the left and right sides for the dorsal and ventral views of the cranium to avoid the effects of bilateral asymmetry. The size of each cranium was estimated using its centroid size, the square root of the sum of the squares of the distances of each landmark from the centroid (Bookstein, 1991). 

### Statistical analysis

Cranium size differences were tested between sexes (Females: n = 52; Males: n = 37), among the four karyotypic blocks (A: n = 25; B: n = 17; C: n = 18; D: n = 29), and among individuals with different karyotypes (2n = 54(A): n = 19; 2n = 54(C): n = 18; 2n = 55a: n = 6; 2n = 55b: n = 5; 2n = 56b: n = 24; 2n = 58: n = 17) using an analysis of variance (ANOVA). The karyotype 2n = 54 occurs in karyotypic blocks A and C, so we named 2n = 54 (A) and 2n = 54 (C), to test for differences between them. Differences in cranium size of specimens from different karyotypic blocks and sexes were visualized through box plots. For multiple comparisons were used Tukey’s test. Differences in cranium shape between sexes, among four karyotypic blocks, and among individuals with different karyotypes as well as their interactions, were tested through multivariate analysis of variance (MANOVA). The Bonferroni correction for multiple comparisons was applied. To test for significant shape differences induced by rearrangements of chromosomal pairs 1 and 2, separate MANOVAs were used. In these two designs, the categories compared were metacentric homozygotes (MM), acrocentric homozygotes (AA), and heterozygotes (MA). The sample size of crania used for each pair was: pair 1 MM n = 43; MA: n = 5; AA: n = 41; and pair 2 MM n = 66; MA: n = 6; AA: n = 17.

Principal component analysis (PCA) used the variance-covariance matrix of generalized least-squares superimposition residuals. PCs of the covariance matrix of superimposition residuals were used as new shape variables in a linear discriminant analysis (LDA - explained below) to reduce the data set’s dimensionality and work on independent variables. The matrices of PCA scores for each view of the cranium were joined in one total matrix, and a subsequent matrix was used for a PCA to pool dorsal, ventral, and lateral information in the same analysis (Cordeiro-Estrela et al., 2006). 

To choose the number of PCs to be included in the linear discriminant analysis (LDA), we computed correct classification percentages with each combination of PCs (Baylac and Friess, 2005). We selected the subset of PCs giving the highest overall good classification percentage. We used a leave-one-out cross-validation procedure that allows an unbiased estimate of classification percentages (Baylac and Friess, 2005). Cross-validation is used to evaluate the performance of classification by LDA. In the leave-one-out cross-validation, all the data except 1 individual are used to calculate the discriminant function. The individual not used is then classified. The procedure is repeated to compute a mean classification error and a probability of group membership for each individual. The visualization of shape differences for the three views of the cranium was obtained through multivariate regression of shape variables discriminant axes. 

### Morphometric and geographic distances

To visualize the morphological relationship among specimens with different karyotypes, Mahalanobis distances were used to compute a neighbor-joining tree. We calculated geographic distances for morphometric data from the cranium among karyotypes and estimated them among each karyotypic block. We used Mantel’s test to evaluate the correlation between morphometric and geographic matrices. The geographic distance matrix is based on the linear distances of each locality calculated by software Geographic Distance Matrix Generator, version 1.2.3 (Ersts, 2009). 

For all statistical analyses and to generate graphics, we used the “R” language and environment for statistical computing version 2.2.1 for Linux (R Development Core Team, 2022; http://www.R-project.org) and the following libraries: MASS (Venables and Ripley, 2002) and APE version 1.8-2 (Paradis et al., 2006). Geometric morphometric procedures were carried out with the Rmorph package (Baylac, 2006), a geometric and multivariate morphometrics library. 

## Results

### Size

**Figure 3 -:**
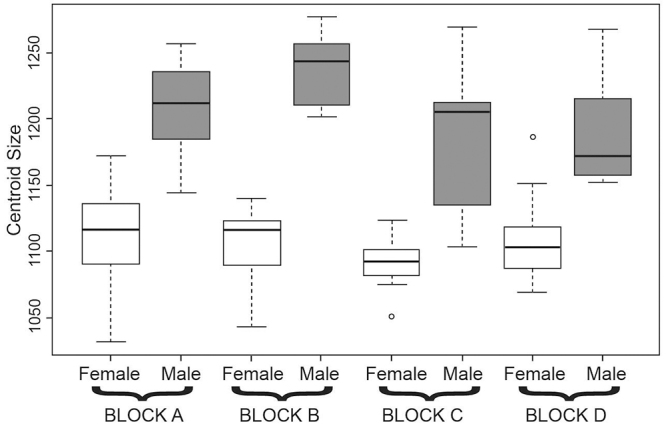
Variation of centroid size for dorsal view of the cranium of *Ctenomys lami* for each sex and karyotypic block. The horizontal line represents the mean; box margins are at 25th and 75th percentiles; bars extend to 5th and 95th percentiles; and circles are outliers. Different colors of boxes represent significant differences for Tukey’s multiple comparison tests at the 5% level.

We found significant differences in centroid size related to sexual dimorphism for dorsal (*F* = 153.5, *P* < 0.001), ventral (*F* = 125.5, *P* < 0.001), and lateral (*F* = 151.1, *P* < 0.001) cranial views. Males are, on average larger than females in all karyotypic blocks (Figure 3). The ANOVAs among four karyotypic blocks, among specimens with different karyotypes, and chromosome pairs (metacentric, acrocentric - homozygotes and heterozygotes: MM, AA and MA) were not significant for centroid size (*P* > 0.05). 

### Shape

**Figure 4 -:**
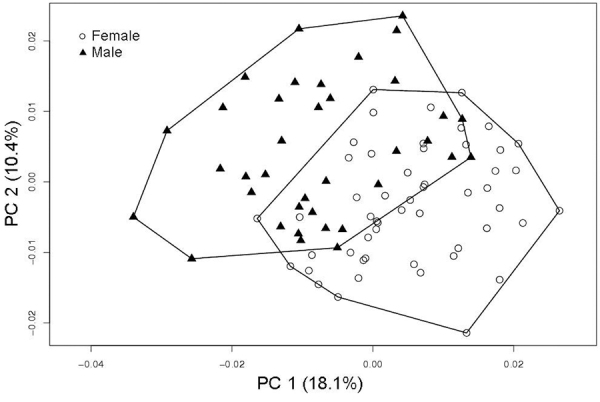
Two first principal components for males and females of *Ctenomys lami* for ventral view of the cranium with percent of variance explained for each principal component.

The interaction factor for MANOVA among factors, sex, karyotypic blocks, karyotypes, and chromosomal rearrangements (chromosome pairs) was not significant for cranium shape (dorsal: Wilks’λ = 0.72, *F* = 0.88, *P* = 0.631; ventral: Wilks’λ = 0.64, *F* = 1.02, *P* = 0.444; lateral: Wilks’λ = 0.46, *F* = 0.88, *P* = 0.682). The first two principal components suggest a differentiation between males and females for the ventral view of the cranium (Figure 4); similar results were found with the other views (complementary results in File S3). The MANOVA results indicate significant sexual dimorphism in shape for the three views of the cranium (dorsal: Wilks’λ = 0.31, *F* = 7.5, *P* < 0.001; ventral: Wilks’λ = 0.36, *F* = 12.4, *P* < 0.001; and lateral: Wilks’λ = 0.22, *F* = 7.8, *P* < 0.001). The correct classification percentage of the discriminant analysis averaged 97.1% for females (min. 94.2%, max. 100%) and 90.5% for males (min. 86.5%, max. 97.3%) for three views of the cranium. 

We found differences in shape between the two major groups, AB and CD. The MANOVA for the three views of the cranium were significant (MANOVA - dorsal: Wilks’λ = 0.45, *F* = 3.84, *P* < 0.001; and ventral: Wilks’λ = 0.44, *F* = 7.8, *P* < 0.001; and lateral: Wilks’λ = 0.55, *F* = 5.2, *P* < 0.001). 

**Figure 5 -:**
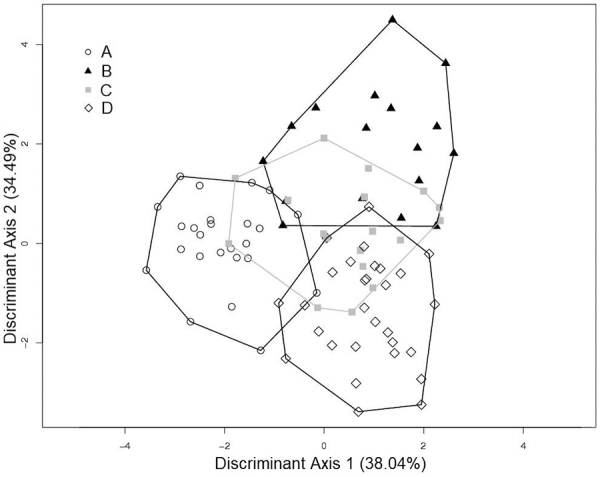
Two first axes of discriminant analysis for karyotypic blocks (A, B, C and D) of *Ctenomys lami* for dorsal, ventral and lateral views of the cranium integrated. The percentage of variance explained for each axis is given in parenthesis.

The cranium of specimens from the four karyotypic blocks differed significantly for the three views of the cranium (MANOVA - dorsal: Wilks’λ = 0.21, *F* = 4.17, *P* < 0.001; ventral: Wilks’λ = 0.13, *F* = 3.82, *P* < 0.001; and lateral: Wilks’λ = 0.28, *F* = 2.43, *P* < 0.001). Pairwise comparisons revealed significant differences among all four karyotypic blocks (Table 1). The higher *F* value was recovered for the A × D comparison, the extremes of the distribution, and the lower *F* value for C × D blocks (Table 1). The discriminant analysis for karyotypic blocks using the three views of the cranium integrated showed three groups separated in mean (blocks A, B, and D) block C, it partially superimposed on the other blocks (Figure 5). The percentual of variance explained for the two first discriminant axes for the three views of the cranium and the views integrated is given in Table 2. The smaller percentage of correct classification were for ventral view of the cranium (75.8%), and the higher percentage was for a dorsal view (85.7%). The karyotypic block that showed the smallest percentage of correct classification is block C (76.3%), and block D have the highest percentage of correct classification (87.0%). 

**Table 1 -:** MANOVA among karyotypic blocks (A, B, C and D) of *Ctenomys lami* for cranial shape (results for dorsal, ventral and lateral views integrated).

Comparison	λ_Wilks_	*F*	*P*
A ? B	0.18	5.71	4.23 × 10^-4^**
A ? C	0.47	7.59	3.23 × 10^-4^**
A ? D	0.42	12.84	4.69 × 10^-7^**
B ? C	0.40	4.48	0.00595**
B ? D	0.28	3.19	0.00818**
C ? D	0.43	2.26	0.0288*

*P < 0.05; **P < 0.01; after Bonferroni correction.

**Table 2 -:** Percent accumulated of variance explained for two first discriminant axes of the discriminant analyses performed for four karyotypic blocks of *Ctenomys lami* for each view of the cranium and three vies integrated.

	% of variance explained	
	Discriminant axis 1	Discriminant axis 1 + axis 2
Dorsal	38.12	71.96
Ventral	45.20	78.26
Lateral	41.98	77.07
3 views	38.04	72.53

For specimens with different karyotypes, we found significant differences in cranium shape (dorsal: Wilks’λ = 0.18, *F* = 2.78, *P* < 0.001; ventral: Wilks’λ = 0.2, *F* = 2.32, *P* < 0.001; and lateral: Wilks’λ = 0.05, *F* = 1.97, *P* < 0.001). Pairwise comparisons among different karyotypes specimens (including the cytotype 2n = 54 that occurs in two blocks, A and C) were significant for six of the 15 comparisons (Table 3). There are significant differences among specimens with karyotypes 2n = 54, 2n = 56b, and 2n = 58; and between 2n = 54 specimens of blocks A and C. On the other hand, the hybrid forms 2n = 55a and 2n = 55b karyotypes were not significantly different in cranium shape from any other karyotypic specimens (Table 3).

**Table 3 -:** MANOVA resume with *F* and *P* values between six different diploid numbers compared pair to pair for cranial shape of *Ctenomys lami* (integrated results for dorsal, ventral and lateral views). Parenthesis indicate the karyotypic block for each karyotype.

	54 (A)	55a (A)	58 (B)	54 (C)	55b (D)
55a (A)	NS	-			
58 (B)	5.0 / 0.01*	NS	-		
54 (C)	4.96 / 0.009**	NS	4.48 / 0.02*	-	
55b (D)	NS	NS	NS	NS	-
56b (D)	7.26 / 0.0003**	NS	5.31 / 0.006**	1.91 / 0.009**	NS

*P < 0.05; **P < 0.01; after Bonferroni correction; NS = statistically not significant.

For specimens whose karyotype showed variation in autosomal complement pair 1 (chromosomal pair 1) showed significant differences for the dorsal view and three views pooled of the cranium. Pairwise comparisons showed significant differences among all rearrangements for pair 1 only for the dorsal view. Metacentric homozygote *versus* heterozygote (MM × MA: Wilks’λ = 0.51, *F* = 2.62, *P* < 0.05); metacentric homozygote *versus* acrocentric homozygote (MM × AA: Wilks’λ = 0.63, *F* = 3.6, *P* < 0.01); and heterozygote *versus* acrocentric homozygote (MA × AA Wilks’λ = 0.78, *F* = 2.8, *P* < 0.05). However, for chromosomal pair 2, we found significant differences only for the comparison between homozygote chromosome forms (MM × AA) for dorsal (Wilks’λ = 0.62, *F* = 5.5, *P* < 0.001), and ventral (Wilks’λ = 0.6, *F* = 4.1, *P* < 0.001) views.

### Morphological description based in landmarks deformations

**Figure 6 -:**
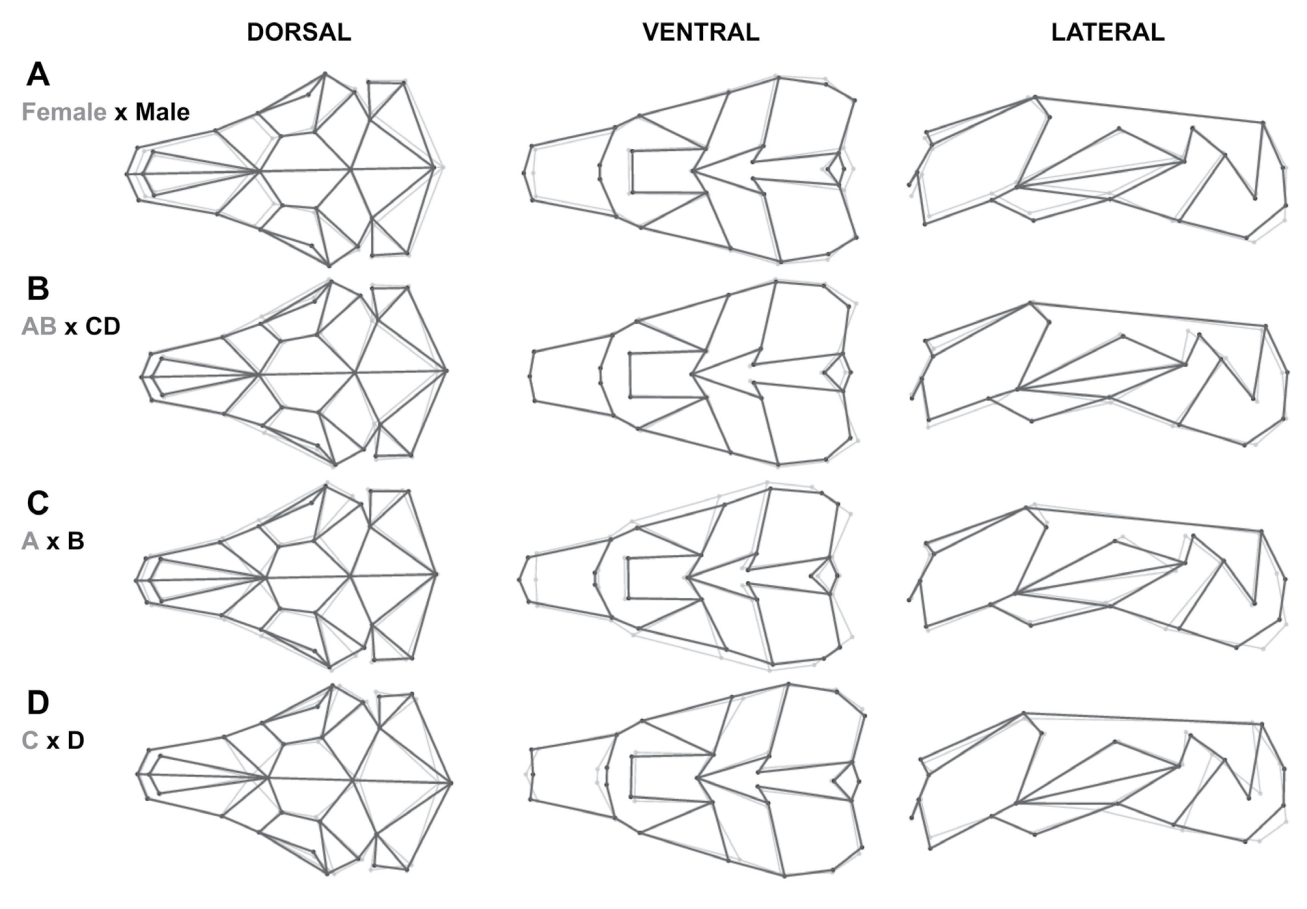
Shape differences in *Ctenomys lami* cranium views (dorsal, ventral, and lateral). A) mean shape differences between sexes, females (gray lines) and males (black lines) specimens. B) mean shape differences between the two great groups, AB (gray lines) and CD (black lines) karyotypic blocks specimens. C) mean shape differences between A (gray lines) and block B (black lines) karyotypic blocks specimens. D) mean shape differences in cranium between C (gray lines) and D (black lines) karyotypic blocks specimens. Shape differences in C and D lines are amplified two times for better visualization.

Males of *C. lami* have a relatively longer and wider rostrum and nasal bone, a wider zygomatic arch, and a jugal bone proportionally higher than females, that have a proportionately larger neurocranial region and a wider tympanic bulla (Figure 6A). The cranium of the major group AB is proportionally wider at the zygomatic arch, and at the external auditory meatus, the rostrum is deeper, and the tympanic bulla is longer in relation to the CD group (Figure 6B). There are also differences in shape between A and B blocks, A has a rostrum and nasals smaller, a wider zygomatic arch, more expanded tympanic bulla, and bigger frontal bones than B (Figure 6C). Between C and D karyotypic blocks, the C block has a smaller nasals, deeper tympanic bulla and longer frontals than the D block (Figure 6D). 

### Morphometric distances

**Figure 7 -:**
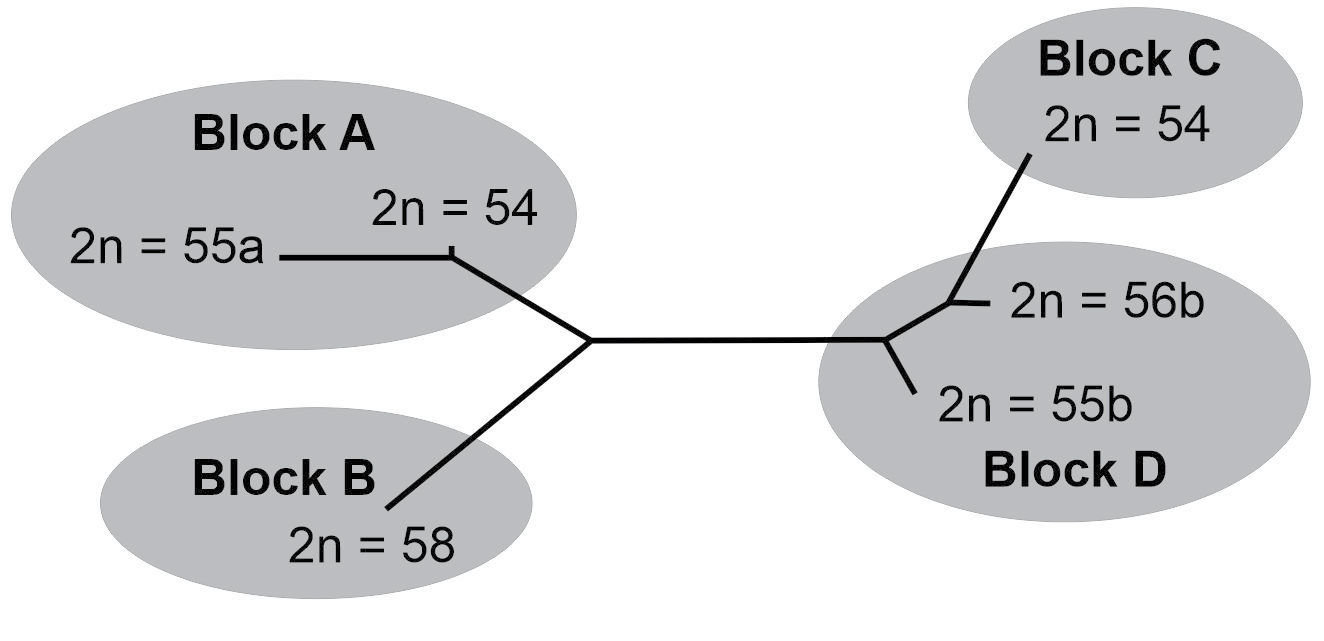
Neighbor-joining phenogram based on the Mahalanobis distances among specimens with different karyotypes for ventral view of the cranium of *Ctenomys lami*. The branches are proportional to morphological distances and ellipses indicate the karyotypic blocks.

For specimens with different karyotypes, we calculated the Mahalanobis distances for cranial shape variation. The Mahalanobis distances for the ventral view of the cranium showed six statistically significant distances after the Bonferroni correction (Table 4). The first significant distance is related to individuals 2n = 54 (C block) and specimens 2n = 55a (A block); the second distance between specimens with 2n = 58 (B) and specimens 2n = 56b (D); the third for specimens 2n = 54 (A) and specimens 2n = 54 (C); the fourth between specimens 2n = 54 (C) and specimens 2n = 58 (B); the fifth for specimens 2n = 54 (A) and specimens 2n = 58 (B); and finally, the sixth between specimens 2n = 54 (A) and specimens 2n = 56b (D). Therefore, the larger distances are among skulls of karyotypic blocks and not within block. The neighbor-joining phenogram in Figure 7 is based on Mahalanobis distances for the ventral view of the cranium and shows the relationship among specimens with different karyotypes. The branches are proportional to Mahalanobis distances. The trees generated for dorsal and lateral views of the cranium do not agree in topology with the phenogram for the ventral view (data not shown). 

**Table 4 -:** The matrix of Mahalanobis distances among species for different karyotypes for cranium shape of *Ctenomys lami* generated by ventral view of the cranium. Parenthesis indicate the karyotypic block for each karyotype.

	54 (A)	55a (A)	58 (B)	54 (C)	55b (D)
55a (A)	1.693	-			
58 (B)	4.634*	5.326	-		
54 (C)	7.351**	8.096**	6.673**	-	
55b (D)	4.746	7.241	5.817	3.325	-
56b (D)	4.632*	7.357	7.806*	2.016	1.22

*P < 0.05; **P < 0.01, after Bonferroni correction.

### Correlation between morphologic and geographic distances

The Mantel’s test showed no significant correlation between morphological and geographic matrices for the three views of the cranium (*P* > 0.8). This result suggests no association between cranial shape and linear distances among specimens. 

## Discussion

We found shape and size cranium variation in *C. lami*, with significant differences detected among geographically close specimens with different karyotypes. This variation was detected within the restricted occurrence region, perhaps the less wide of a *Ctenomys* species, with a distribution range of 78 by 12 km, approximately 940 km^2^. This intraspecific morphological differentiation followed some patterns, detailed below.

### Sexual dimorphism in cranium

The sexual size dimorphism for the *Ctenomys* genus is well known (Pearson, 1959; Pearson *et al*., 1968; Cook et al., 1990; Freitas, 1990; Malizia and Busch, 1991; Gastal, 1994; Zenuto and Busch, 1998; Marinho and Freitas, 2006). Usually, sexual dimorphism in size is associated with sexual selection or niche divergence within species (Hood, 2000). El Jundi and Freitas (2004) suggest that this dimorphism in *C. lami* might be associated with competition for resources and/or reproduction. Our results also showed a strong sexual dimorphism in the cranium shape, probably related to the same factors as size sexual dimorphism. The correct classification percentage was higher in females than males. These can be due to differences in sample size, with more females than males.

### Karyotypic blocks and cranium morphology

The *C. lami* cranium shape differed between two major groups (blocks A and B and blocks C and D) in the middle of the species distribution (Figure 1). Moreira et al. (1991), using biochemical polymorphisms, showed differences between the two major groups, 1 (AB) and 2 (CD), that are separated by a physical barrier, the connection of two swamps (see the map in Figure 1). However, the biochemical results also indicated gene flow among adjacent subpopulations. Therefore, in dry seasons the natural barrier may weaken or disappear and thus permit gene flow. In the same way, the phenogram based on Mahalanobis distances for the ventral view of the cranium shows a great distance between blocks AB and blocks CD (Figure 7). The trees for morphological distances for the other views of the cranium (dorsal, lateral, and three integrated views) did not show the same pattern (data not shown). 

For the validity of four karyotypic blocks proposed by Freitas (1990, 2007), our results agree when comparing blocks pair to pair. The hypothesis of four blocks is related to the frequency of different karyotypes found for *C. lami* and the topography of the region where they live. On the other hand, El Jundi (2003) did not find consistency between microsatellite DNA data and karyotypic blocks of *C. lami*. Nevertheless, Freitas (1990, 2007) found hybrid forms between A and B blocks (2n = 57 result from 2n = 56a × 2n = 58) and between C and D blocks (2n = 55b result from 2n = 54 × 2n = 56b) but not between B and C blocks; which reinforce the hypothesis of two major groups. Freitas (2007), using chromosome polymorphism found high *F*_*ST*_ values that indicate very low gene flow among blocks, except for the hybrid zones, and suggested a well-defined population structure in four karyotypic blocks.

### Chromosome numbers and geographic variation

Comparisons among cranium shapes for different karyotypes also agree with the subdivision in four-blocks subdivision. Because significant differences were found only in karyotypes from different karyotypic blocks but never in the same block. We did not find differences between hybrid karyotypes 2n = 55a and 2n = 55b with other chromosome forms, possibly due to a reduced sample of two hybrid cytotypes or hybrids having a cranium morphology more similar to the parent form. Marinho and Freitas (2000) studied craniometric variation in *C. minutus* with linear morphometrics and found that hybrid form 2n = 47 was more similar to 2n = 48 than 2n = 46. This species is very similar to *C. lami* (Freitas, 2001, 2006), and the same pattern can occur in *C. lami*. 

The specimens from localities within karyotypic block C have an intermediate cranium shape pattern related to the other blocks in discriminant analysis (Figure 5). In this block, a great frequency of 2n = 54 is probably the ancestral karyotype in the Coxilha da Lombas region (Freitas, 1990). Thus, the earliest populations of *C. lami* could be 2n = 54 and spread along to actual distribution and, in time, accumulate new chromosome rearrangements and different morphological traits, at least in the cranium. Nowadays, gene flow permits hybrids occurrence and this pattern of cranium shape variation. 

Freitas (2006) affirms that in *C. torquatus*, the karyotype 2n = 46 is derived from 2n = 44, and in *C. lami*, this pattern of chromosomal evolution than a smaller diploid number to originate a bigger one can be found. In *C. lami*, the diploid number 2n = 58 specimens appear to be more different in cranium morphology than in any other chromosome population. The most frequent karyotype is 2n = 54 (n = 37), and the most derived is 2n = 58 (n = 17), so the more derived in karyotype is also derived in cranium shape. This does not necessarily indicate an association between chromosomal and morphological evolution. Besides, the two karyotypic blocks had the same 2n = 54 karyotype (A and C blocks), but their cranium shapes differed. Therefore, although they coincide with the geographic distribution, no other evidence would permit us to suggest that the chromosomal alterations directly influence the alteration in the cranium shape in *C. lami*.

Márquez et al. (2000) suggest an association in cranial variation with karyotypic differences for *Nephelomys albigularis*, as synonym of *Oryzomys albigularis* like cited by Márquez *et al*. (2000) with linear morphometrics. Nevertheless, Chondropoulos et al. (1996) proposed that morphological variation is associated with geographical and not karyotype in *Mus musculus*. In opposition Corti and Rohlf (2001) found differences in cranium shape among chromosomal races of *Mus musculus domesticus* using a geometric morphometric approach. Therefore, chromosomal and morphological evolution seems have played an important role in the speciation of the genus *Ctenomys*, but those events must have occurred independently. El Jundi and Freitas (2004) propose the occurrence of genetic drift due to *C. lami* present genetic and demographic patterns indicating a species with little movement and a low genetic flow that turn small populations isolated. Lopes and Freitas (2012) found a pattern of a cline in the stepping-stone model for the genetic variation of *C. lami*. We did not find a correlation between morphological and geographic distances. Thus, our data do not support the hypothesis of morphological cline pattern in cranium shape in an almost linear distribution of *C. lami*. These do not indicate a constant gene flow among small populations but a certain constraint among a few. In the same way, D’Anatro and Lessa (2006) found no correlation between geographic and morphological distances for *C. rionegrensis* using cranium geometric morphometrics. In the same way, our results do not support a direct relationship between chromosomal variation and cranial morphological variation in *C. lami*, corroborating both interspecific (Fernandes et al., 2009; Fornel et al., 2010) and intraspecific data for the genus *Ctenomys* (Fornel *et al*., 2018).

### Cranial variation and chromosome rearrangements

For chromosome rearrangements in pair 1, all comparisons between specimens with metacentric and acrocentric forms, homozygote, or heterozygote, showed differences in cranium shape only for the dorsal view. This can be due dorsal view having more variation than other views of the cranium, as seen in *C. minutus* (Fornel et al., 2010). For chromosomal pair 2 rearrangements, one comparison showed a difference in cranium shape, the comparison between homozygotes forms (metacentric *versus* acrocentric). These because specimens from the double metacentric from A and D karyotypic blocks and the double acrocentric is formed for B block specimens. Therefore, more evidence reinforces differences in cranium shape among karyotypic blocks. 

## Conclusions

The genus *Ctenomys* has cranium adaptations for chisel-tooth digger (Vassallo 1998, 2002; Verzi and Olivares, 2006; Vassallo *et al*., 2021), especially the angle of incisor procumbency related to a longer rostrum (Mora et al., 2003; Vassallo and Mora, 2007). Our results show that *C. lami* specimens of the major group AB have a deeper and stronger rostrum and a larger zygomatic arch than CD karyotypic blocks (Figure 6B), this difference could be adaptative because AB karyotypic blocks with more robust cranium live in a region far away from the coast in relation to CD karyotypic blocks that are closer to sandy soils. A similar pattern is found in related species *Ctenomys minutus* (Kubiak et al., 2018; Galiano and Kubiak, 2021). However, this pattern is not confirmed when comparing only the C block with the D block. A possible explanation could be the fact that *C. lami* occurs in a narrow range without environmental gradient and, thus, without morphological gradient.

In conclusion, *C. lami* has a high chromosomal variation and high cranium morphological variation in a small area of occurrence. We found that morphological differences are related to chromosome numbers and karyotypic blocks, showing for the first time that an association between morphology and chromosome configuration exists on such a very small spatial scale. It is still an open question if the pattern of cranium shape variation is a case of divergent evolution or polymorphism.

## Supplementary material

The following online material is available for this article:

Table S1 - Specimens examined.

File S1 - Detailed list of examined specimens.

File S2 - Definition of landmarks.

File S3 - Complementary results.
