# Supplementary material for: Cranial morphological variation of Ctenomys lami (Rodentia: Ctenomyidae) in a restricted geographical distribution
Source: Genet Mol Biol. 2023 Nov 13;46(3 Suppl 1):e20230130. doi: 10.1590/1678-4685-GMB-2023-0130 (PMC10655944; doi:10.1590/1678-4685-GMB-2023-0130)
Supplement: Table S1 - [file 1415-4757-GMB-46-3-s1-e20230130-s1.pdf]

Genetics and Molecular Biology
https://doi.org/10.1590/1678-4685-GMB-2023-0130

Supplementary Material to "Cranial morphological variation of *Ctenomys lami* (Rodentia: Ctenomyidae) in a restricted geographical distribution"

Table S1 - Number of specimens examined of *Ctenomys lami.*

Population block	Number of collection site*	Karyotype (2n)	Year of collection	*N* males	*N* females	*N* total
**A**	1	54/55a	1981/82 /83	5	11	16
	4	54/55a	1984	4	2	6
	10	54	1984	1	2	3
**B**	5	58	1984	2	3	5
	6	58	1984	-	1	1
	7	58	1984	1	-	1
	8	58	1984	1	-	1
	11	58	1984	-	3	3
	12	58	1983/85	2	2	4
	13	58	1983	2	-	2
**C**	2	54	1984	3	2	5
	3	54	1984	2	4	6
	14	54	1983/85	1	3	4
	17	54	1985	3	-	3
**D**	9	55b	1992	1	1	2
	15	56b	1983/84/85	3	8	11
	16	56b	1983/90	1	4	5
	18	55b/56b	1985	5	6	11
				37	52	89

* See the map in Fig. 1 for details.
