# Supplementary material for: Cranial morphological variation of Ctenomys lami (Rodentia: Ctenomyidae) in a restricted geographical distribution
Source: Genet Mol Biol. 2023 Nov 13;46(3 Suppl 1):e20230130. doi: 10.1590/1678-4685-GMB-2023-0130 (PMC10655944; doi:10.1590/1678-4685-GMB-2023-0130)
Supplement: File S1 - [file 1415-4757-GMB-46-3-s1-e20230130-s2.pdf]

Genetics and Molecular Biology
https://doi.org/10.1590/1678-4685-GMB-2023-0130

Supplementary Material to "Cranial morphological variation of *Ctenomys lami* (Rodentia: Ctenomyidae) in a restricted geographical distribution"

File S1 - List of specimens examined of *Ctenomys lami*. All specimens from the same mammal collection: Coleção de Mamíferos do Departamento de Genética (CMG) da Universidade Federal do Rio Grande do Sul (UFRGS), Porto Alere, RS, Brazil. Each specimen with collection number, sex, diploid number, population block, locality number (see the map in Figure 1). In parentheses latitude and longitude coordinates of each site of collection.

CMG.0009, female, 2n=56b, Block D, locality 18 (29°58'37.10"S, 50°34'12.87"W)

CMG.0010, female, 2n=56b, Block D, locality 18 (29°58'37.10"S, 50°34'12.87"W)

CMG.0011, female, 2n=55b, Block D, locality 18 (29°58'37.10"S, 50°34'12.87"W)

CMG.0012, female, 2n=56b, Block D, locality 18 (29°58'37.10"S, 50°34'12.87"W)

CMG.0013, female, 2n=55b, Block D, locality 18 (29°58'37.10"S, 50°34'12.87"W)

CMG.0014, female, 2n=56b, Block D, locality 18 (29°58'37.10"S, 50°34'12.87"W)

CMG.0015, female, 2n=56b, Block D, locality 18 (29°58'37.10"S, 50°34'12.87"W)

CMG.0016, male, 2n=55b, Block D, locality 18 (29°58'37.10"S, 50°34'12.87"W)

CMG.0017, male, 2n=56b, Block D, locality 18 (29°58'37.10"S, 50°34'12.87"W)

CMG.0018, male, 2n=56b, Block D, locality 18 (29°58'37.10"S, 50°34'12.87"W)

CMG.0019, male, 2n=56b, Block D, locality 18 (29°58'37.10"S, 50°34'12.87"W)

CMG.0182, female, 2n=58, Block B, locality 6 (30°16'29.32"S, 50°57'18.49"W)

CMG.0194, female, 2n=54, Block A, locality 10 (30°18'42.79"S, 50°57'33.85"W)

CMG.0196, female, 2n=58, Block B, locality 11 (30°17'38.10"S, 50°56'28.13"W)

CMG.0197, female, 2n=58, Block B, locality 11 (30°17'38.10"S, 50°56'28.13"W)

CMG.0202, male, 2n=58, Block B, locality 8 (30°16'46.44"S, 50°56'21.01"W)

CMG.0204, male, 2n=54, Block C, locality 17(29°58'38.05"S, 50°36'30.34"W)

CMG.0206, male, 2n=54, Block A, locality 10 (30°18'42.79"S, 50°57'33.85"W)

CMG.0207, male, 2n=58, Block B, locality 7 (30°16'05.33"S, 50°55'46.86"W)

CMG.0208, female, 2n=58 Block B, locality 11 (30°17'38.10"S, 50°56'28.13"W)

CMG.0210, female, 2n=54, Block A, locality 10 (30°18'42.79"S, 50°57'33.85"W)

CMG.0364, male, 2n=56b, Block D, locality 16 (29°58'15.69"S, 50°35'38.03"W)

CMG.0454, female, 2n=55b, Block D, locality 9 (29°56'01.71"S, 50°28'20.00"W)

CMG.0457, male, 2n=55b, Block D, locality 9 (29°56'01.71"S, 50°28'20.00"W)

CMG.0501, male, 2n=58, Block B, locality 5 (30°16'25.89"S, 50°58'15.14"W)

CMG.0503, male, 2n=54, Block A, locality 1 (30°16'13.21"S, 50°00'49.95"W)

CMG.0504, female, 2n=54, Block C, locality 14 (30°05'54.71"S, 50°44'58.70"W)

CMG.0505, male, 2n=54, Block A, locality 4 (30°18'42.92"S, 50°57'24.43"W)

CMG.0506, female, 2n=56b, Block D, locality 16 (29°58'15.69"S, 50°35'38.03"W)

CMG.0507, male, 2n=55a, Block A, locality 1 (30°16'13.21"S, 50°00'49.95"W)

CMG.0508, female, 2n=54, Block C, locality 14 (30°05'54.71"S, 50°44'58.70"W)

CMG.0509, female, 2n=54, Block C, locality 3 (30°01'29.37"S, 50°39'20.77"W)

CMG.0510, female, 2n=54, Block A, locality 4 (30°18'42.92"S, 50°57'24.43"W)

CMG.0511, male, 2n=58, Block B, locality 13 (30°17'57.83"S, 50°50'13,72"W)

CMG.0513, male, 2n=58, Block B, locality 5 (30°16'25.89"S, 50°58'15.14"W)

CMG.0514, female, 2n=55a, Block A, locality 4 (30°18'42.92"S, 50°57'24.43"W)

CMG.0515, female, 2n=54, Block C, locality 14 (30°05'54.71"S, 50°44'58.70"W)

CMG.0516, male, 2n=58, Block B, locality 13 (30°17'57.83"S, 50°50'13,72"W)

CMG.0517, male, 2n=54, Block A, locality 1 (30°16'13.21"S, 50°00'49.95"W)

CMG.0518, male, 2n=54, Block A, locality 1 (30°16'13.21"S, 50°00'49.95"W)

CMG.0519, male, 2n=54, Block A, locality 4 (30°18'42.92"S, 50°57'24.43"W)

CMG.0520, female, 2n=56b, Block D, locality 15 (29°58'25.42"S, 50°35'26.47"W)

CMG.0521, male, 2n=58, Block B, locality 12 (30°08'35.48"S, 50°51'21.45"W)

CMG.0522, female, 2n=56b, Block D, locality 15 (29°58'25.42"S, 50°35'26.47"W)

CMG.0523, male, 2n=54, Block C, locality 17(29°58'38.05"S, 50°36'30.34"W)

CMG.0524, female, 2n=56b, Block D, locality 16 (29°58'15.69"S, 50°35'38.03"W)

CMG.0526, Male, 2n=56b, Block D, locality 15 (29°58'25.42"S, 50°35'26.47"W)

CMG.0530, female, 2n=56b, Block D, locality 15 (29°58'25.42"S, 50°35'26.47"W)

CMG.0531, female, 2n=58, Block B, locality 12 (30°08'35.48"S, 50°51'21.45"W)

CMG.0534, male, 2n=54, Block A, locality 4 (30°18'42.92"S, 50°57'24.43"W)

CMG.0535, male, 2n=54, Block C, locality 17(29°58'38.05"S, 50°36'30.34"W)

CMG.0536, male, 2n=58, Block B, locality 12 (30°08'35.48"S, 50°51'21.45"W)

CMG.0538, male, 2n=54, Block C, locality 14 (30°05'54.71"S, 50°44'58.70"W)

CMG.0540, female, 2n=56b, Block D, locality 16 (29°58'15.69"S, 50°35'38.03"W)

CMG.0549, male, 2n=55a, Block A, locality 1 (30°16'13.21"S, 50°00'49.95"W)

CMG.0621, female, 2n=55a, Block A, locality 1 (30°16'13.21"S, 50°00'49.95"W)

CMG.2905, female, 2n=54, Block A, locality 1 (30°16'13.21"S, 50°00'49.95"W)

CMG.2906, female, 2n=54, Block A, locality 1 (30°16'13.21"S, 50°00'49.95"W)

CMG.2908, female, 2n=54, Block A, locality 1 (30°16'13.21"S, 50°00'49.95"W)

CMG.2909, female, 2n=54, Block A, locality 1 (30°16'13.21"S, 50°00'49.95"W)

CMG.2910, female, 2n=54, Block A, locality 1 (30°16'13.21"S, 50°00'49.95"W)

CMG.2912, female, 2n=54, Block A, locality 1 (30°16'13.21"S, 50°00'49.95"W)

CMG.2914, female, 2n=54, Block A, locality 1 (30°16'13.21"S, 50°00'49.95"W)

CMG.2915, female, 2n=54, Block A, locality 1 (30°16'13.21"S, 50°00'49.95"W)

CMG.2916, male, 2n=54, Block C, locality 3 (30°01'29.37"S, 50°39'20.77"W)

CMG.2917, male, 2n=54, Block C, locality 3 (30°01'29.37"S, 50°39'20.77"W)

CMG.2918, female, 2n=55a, Block A, locality 1 (30°16'13.21"S, 50°00'49.95"W)

CMG.2919, female, 2n=54, Block A, locality 1 (30°16'13.21"S, 50°00'49.95"W)

CMG.2920, male, 2n=56b, Block D, locality 15 (29°58'25.42"S, 50°35'26.47"W)

CMG.2021, female, 2n=58, Block B, locality 5 (30°16'25.89"S, 50°58'15.14"W)

CMG.2922, male, 2n=54, Block C, locality 2 (30°00'29.89"S, 50°38'33.50"W)

CMG.2923, female, 2n=56b, Block D, locality 15 (29°58'25.42"S, 50°35'26.47"W)

CMG.2924, female, 2n=56b, Block D, locality 15 (29°58'25.42"S, 50°35'26.47"W)

CMG.2925, female, 2n=58, Block B, locality 12 (30°08'35.48"S, 50°51'21.45"W)

CMG.2926, male, 2n=54, Block C, locality 2 (30°00'29.89"S, 50°38'33.50"W)

CMG.2927, female, 2n=54, Block C, locality 3 (30°01'29.37"S, 50°39'20.77"W)

CMG.2928, female, 2n=54, Block C, locality 2 (30°00'29.89"S, 50°38'33.50"W)

CMG.2929, male, 2n=54, Block C, locality 2 (30°00'29.89"S, 50°38'33.50"W)

CMG.2930, female, 2n=54, Block C, locality 2 (30°00'29.89"S, 50°38'33.50"W)

CMG.2931, female, 2n=56b, Block D, locality 15 (29°58'25.42"S, 50°35'26.47"W)

CMG.2932, female, 2n=56b, Block D, locality 15 (29°58'25.42"S, 50°35'26.47"W)

CMG.2934, female, 2n=54, Block C, locality 3 (30°01'29.37"S, 50°39'20.77"W)

CMG.2935, female, 2n=56b, Block D, locality 15 (29°58'25.42"S, 50°35'26.47"W)

CMG.2936, female, 2n=58, Block B, locality 5 (30°16'25.89"S, 50°58'15.14"W)

CMG.2937, female, 2n=56b, Block D, locality 15 (29°58'25.42"S, 50°35'26.47"W)

CMG.2938, female, 2n=54, Block C, locality 3 (30°01'29.37"S, 50°39'20.77"W)

CMG.2939, female, 2n=58, Block B, locality 5 (30°16'25.89"S, 50°58'15.14"W)

CMG.2998, male, 2n=56b, Block D, locality 15 (29°58'25.42"S, 50°35'26.47"W)

CMG.3226, male, 2n=55a, Block A, locality 4 (30°18'42.92"S, 50°57'24.43"W)
