# Supplementary material for: Cranial morphological variation of Ctenomys lami (Rodentia: Ctenomyidae) in a restricted geographical distribution
Source: Genet Mol Biol. 2023 Nov 13;46(3 Suppl 1):e20230130. doi: 10.1590/1678-4685-GMB-2023-0130 (PMC10655944; doi:10.1590/1678-4685-GMB-2023-0130)
Supplement: File S2 - [file 1415-4757-GMB-46-3-s1-e20230130-s3.pdf]

Genetics and Molecular Biology
https://doi.org/10.1590/1678-4685-GMB-2023-0130

Supplementary Material to "Cranial morphological variation of *Ctenomys lami* (Rodentia: Ctenomyidae) in a restricted geographical distribution"

File S2 - Definition of landmarks with numbers and locations for each view of the cranium of *Ctenomys lami* (represented in Figure 2) based on Fernandes *et al.*, (2009).

Dorsal view: 1. anterior tip of the suture between premaxilla; 2-3. anterolateral extremity of incisive alveolus; 4. anterior extremity of the suture between nasals; 5-6. most anterior point of the suture between nasals and premaxilla; 7-8. most anterior point of the root of zygomatic arch; 9. suture between nasals and frontals; 10-11. anterolateral extremity of lacrimal; 12-13. point of minor breadth between frontals; 14-15. terminal extremity of postorbital process; 16-17. anterior extremity of the suture between frontal and squamosal; 18-19. posterolateral extremity of postorbital process; 20-21. tip of posterior process of jugal; 22. suture between frontals and parietals; 23-24. anterolateral extremity of the suture between parietal and squamosal; 25-26. anterior tip of the external auditory meatus; 27-28. point of maximum curvature on mastoid apophysis; 29. most posterior point of occipital.

Ventral view: 1. anterior tip of the suture between premaxilla; 2-3. anterolateral extremity of incisive alveolus; 4-5. lateral edge of incisive foramen in suture between premaxilla and maxilla; 6-7. most anterior point of the root of zygomatic arch; 8-9. most anterior point of the orbit; 10-11. most anterior point of premolar alveolus; 12-13. posterior extremity of III molar alveolus; 14. posterior extremity of suture between palatines; 15-16. most anterior point of intersection between jugal and squamosal; 17-18. most posterior point of pterygoid; 19-20. anterior extremity of tympanic bulla; 21-22. anterior tip of the external auditory meatus; 23-24. posterior extremity of mastoid apophysis; 25-26. posterior extremity of

paraoccipital process; 27. most anterior point of foramen magnum; 28-29. posterior extremity of occipital condyle; 30. most posterior point of foramen magnum.

Lateral view: 1. most anterior point of premaxilla; 2. most posterior point of incisive alveolus; 3. most inferior point of incisive alveolus; 4. anterior tip of nasal; 5. most anterior point of the suture between nasal and premaxilla; 6. suture between premaxilla, maxilla and frontal; 7. most inferior point of suture between lacrimal and maxilla; 8. most inferior point of infraorbital foramen; 9. most inferior point of suture between premaxilla and maxilla; 10. most anterior point of premolar alveolus; 11. superior extremity of postorbital process; 12. inferior extremity of jugal process; 13. tip of posterior process of jugal; 14. medial point of suture between parietal and squamosal; 15. superior extremity of lambdoidal crest; 16. most superior point of suture between squamosal and tympanic bulla; 17. most posterior point of pterygoid; 18. inferior extremity of mastoid process; 19. most anterior margin of paraoccipital process; 20. most posterior margin of paraoccipital process; 21. posterior extremity of intersection between occipital and tympanic bulla.
