# Supplementary material for: Cranial morphological variation of Ctenomys lami (Rodentia: Ctenomyidae) in a restricted geographical distribution
Source: Genet Mol Biol. 2023 Nov 13;46(3 Suppl 1):e20230130. doi: 10.1590/1678-4685-GMB-2023-0130 (PMC10655944; doi:10.1590/1678-4685-GMB-2023-0130)
Supplement: File S3 - [file 1415-4757-GMB-46-3-s1-e20230130-s4.pdf]

Genetics and Molecular Biology
https://doi.org/10.1590/1678-4685-GMB-2023-0130

Supplementary Material to "Cranial morphological variation of *Ctenomys lami* (Rodentia: Ctenomyidae) in a restricted geographical distribution"

File S3 - Complementary results for all analysis with cranium of *Ctenomys lami*.

den Df = denominator degrees of freedom

Df = degrees of freedom

LDA = least discriminant analysis

Mean Sq = Mean of squares

n = cranium sample size among parenthesis

num Df = numerator degrees of freedom

Sum Sq = Sum of squares

Wilks = Wilks'λ

Size

ANOVAs for cranium centroid size

Factor sex (Females: n = 52; and Males: n = 37)

Cranium Size Dorsal – ANOVA for "sex"

	Df	Sum Sq	Mean Sq	F value	Pr(>F)
sex	1	208657	208657	153.46	< 2.2e-16 ***
Residuals	87	118293	1360		

Cranium Size Ventral – ANOVA for "sex"

	Df	Sum Sq	Mean Sq	F value	Pr(>F)
sex	1	215807	215807	125.86	< 2.2e-16 ***
Residuals	87	149175	1715		

Cranium Size Lateral – ANOVA for "sex"

	Df	Sum Sq	Mean Sq	F value	Pr(>F)
sex	1	201825	201825	151.09	< 2.2e-16 ***
Residuals	87	116213	1336		

Factor karyotypic block (A: n = 25; B: n = 17; C: n = 18; and D: n = 29)

Cranium Size Dorsal – ANOVA for "karyotypic block"

	Df	Sum Sq	Mean Sq	F value	Pr(>F)
block	3	10601	3534	0.9494	0.4205
Residuals	85	316349	3722		

Cranium Size Ventral – ANOVA for "karyotypic block"

	Df	Sum Sq	Mean Sq	F value	Pr(>F)
block	3	14001	4667	1.1302	0.3415
Residuals	85	350982	4129		

Cranium Size Lateral – ANOVA for "karyotypic block"

	Df	Sum Sq	Mean Sq	F value	Pr(>F)
block	3	14363	4788	1.3401	0.2668
Residuals	85	303674	3573		

Factor karyotypic number (2n = 54(A): n = 19; 2n = 2n = 54(C): n = 18; 2n = 55a: n = 6; 2n = 55b: n = 5; 2n = 56b: n = 24; 2n = 58: n = 17)

Cranium Size Dorsal – ANOVA for "karyotypic number"

	Df	Sum Sq	Mean Sq	F value	Pr(>F)
karn	5	10678	2136	0.5604	0.73
Residuals	83	316272	3811		

Cranium Size Ventral – ANOVA for "karyotypic number"

	Df	Sum Sq	Mean Sq	F value	Pr(>F)
karn	5	14119	2824	0.668	0.6488
Residuals	83	350863	4227		

Cranium Size Lateral – ANOVA for "karyotypic number"

	Df	Sum Sq	Mean Sq	F value	Pr(>F)
karn	5	14513	2903	0.7937	0.5572
Residuals	83	303525	3657		

Shape

MANOVAs for cranium shape

Factor sex (Females: n = 52; and Males: n = 37)

Cranium Shape Dorsal – MANOVA for “sex”

	Df	Wilks	approx F	num Df	den Df	Pr(>F)
sex	1	0.3128	7.4710	20	68	1.498e-10 ***
Residuals	87					

Cranium Shape Ventral – MANOVA for “sex”

	Df	Wilks	approx F	num Df	den Df	Pr(>F)
sex	1	0.3612	12.3792	11	77	4.743e-13 ***
Residuals	87					

Cranium Shape Lateral – MANOVA for “sex”

	Df	Wilks	approx F	num Df	den Df	Pr(>F)
sex	1	0.2249	7.7871	27	61	1.925e-11 ***
Residuals	87					

Factor karyotypic block (A: n = 25; B: n = 17; C: n = 18; and D: n = 29)

Dorsal: Wilks’λ = 0.21, F = 4.17, P < 0.001

Ventral: Wilks’λ = 0.13, F = 3.82, P < 0.001

Lateral: Wilks’λ = 0.28, F = 2.43, P < 0.001

Factor larger groups (AB: n = 42; CD: n = 47)

Cranium Shape Dorsal – MANOVA for “larger groups”

	Df	Wilks	approx F	num Df	den Df	Pr(>F)

	Df	Wilks	approx F	num Df	den Df	Pr(>F)
mgroup	1	0.4537	3.8417	21	67	1.379e-05 ***
Residuals	87					

Cranium Shape Ventral – MANOVA for "larger groups"

	Df	Wilks	approx F	num Df	den Df	Pr(>F)
mgroup	1	0.4472	7.8295	12	76	2.891e-09 ***
Residuals	87					

Cranium Shape Lateral – MANOVA for "larger groups"

	Df	Wilks	approx F	num Df	den Df	Pr(>F)
mgroup	1	0.5505	5.1711	12	76	2.933e-06 ***
Residuals	87					

Factor karyotypic number (2n = 54(A): n = 19; 2n = 2n = 54(C): n = 18; 2n = 55a: n = 6; 2n = 55b: n = 5; 2n = 56b: n = 24; 2n = 58: n = 17)

Cranium Shape Dorsal – MANOVA for "karyotypic number"

	Df	Wilks	approx F	num Df	den Df	Pr(>F)
karn	5.00	0.17986	2.78556	55.00	341.49	8.4e-09 ***
Residuals	83.00					

Cranium Shape Ventral – MANOVA for "karyotypic number"

	Df	Wilks	approx F	num Df	den Df	Pr(>F)
karn	5.00	0.20087	2.32320	60.00	340.93	1.219e-06 ***
Residuals	83.00					

Cranium Shape Lateral – MANOVA for "karyotypic number"

	Df	Wilks	approx F	num Df	den Df	Pr(>F)
karn	5.00	0.05066	1.96833	125.00	295.29	1.47e-06 ***
Residuals	83.00					

Factor larger groups (AB: n = 42; CD: n = 47)

Cranium Shape Dorsal – MANOVA for "larger groups"

	Df	Wilks	approx F	num Df	den Df	Pr(>F)
groups	1	0.4537	3.8417	21	67	1.379e-05 ***

Residuals	87					

Cranium Shape Ventral – MANOVA for "larger groups"

	Df	Wilks	approx F	num Df	den Df	Pr(>F)
groups	1	0.4472	7.8295	12	76	2.891e-09 ***
Residuals	87					

Cranium Shape Lateral – MANOVA for "larger groups"

	Df	Wilks	approx F	num Df	den Df	Pr(>F)
groups	1	0.5505	5.1711	12	76	2.933e-06 ***
Residuals	87					

Factor pair 1 and pair 2.

Cranium Shape Dorsal – MANOVA for "pair 1" and "pair 2"

	Df	Wilks	approx F	num Df	den Df	Pr(>F)
Pair1	2	0.46639	2.35460	28	142	0.0005659 ***
Pair2	2	0.47867	2.25869	28	142	0.0010055 **
Residuals	84					

Cranium Shape Ventral – MANOVA for "pair 1" and "pair 2"

	Df	Wilks	approx F	num Df	den Df	Pr(>F)
Pair1	2	0.70798	1.04384	26	144	0.4158910
Pair2	2	0.46341	2.59749	26	144	0.0001797 ***
Residuals	84					

Cranium Shape Lateral – MANOVA for "pair 1" and "pair 2"

	Df	Wilks	approx F	num Df	den Df	Pr(>F)
Pair1	2	0.37289	1.62057	48	122	0.018057 *
Pair2	2	0.31501	1.98682	48	122	0.001363 **
Residuals	84					

Cranium Shape Dorsal - MANOVA Interaction test among factors

	Df	Wilks	approx F	num Df	den Df	Pr(>F)

sex	1.00	0.2664	8.5369	20.00	62.00	2.906e-11 ***
block	3.00	0.0963	3.6879	60.00	185.81	6.013e-12 ***
sex:block	3.00	0.3741	1.2089	60.00	185.81	0.1707
Residuals	81.00					

Cranium Shape Ventral - MANOVA Interaction test among factors

	Df	Wilks	approx F	num Df	den Df	Pr(>F)
sex	1.00	0.3162	13.9574	11.00	71.00	8.581e-14 ***
block	3.00	0.2459	3.8789	33.00	209.88	9.876e-10 ***
sex:block	3.00	0.6718	0.9195	33.00	209.88	0.598
Residuals	81.00					

Cranium Shape Lateral - MANOVA Interaction test among factors

	Df	Wilks	approx F	num Df	den Df	Pr(>F)
sex	1.00	0.1909	8.6335	27.00	55.00	1.094e-11 ***
block	3.00	0.0954	2.4370	81.00	165.38	6.981e-07 ***
sex:block	3.00	0.2119	1.3881	81.00	165.38	0.05945
Residuals	81.00					

Cranium Shape Dorsal - MANOVA Interaction test among factors

	Df	Wilks	approx F	num Df	den Df	Pr(>F)
karn	5.00	0.1571	2.8038	55.00	313.71	9.599e-09 ***
sex	1.00	0.3568	10.9805	11.00	67.00	2.966e-11 ***
karn:sex	5.00	0.4145	1.1952	55.00	313.71	0.1769
Residuals	77.00					

Cranium Shape Ventral - MANOVA Interaction test among factors

	Df	Wilks	approx F	num Df	den Df	Pr(>F)
karn	5.00	0.1683	2.4150	60.00	312.83	4.976e-07 ***
sex	1.00	0.3023	12.6966	12.00	66.00	6.304e-13 ***
karn:sex	5.00	0.4639	0.9293	60.00	312.83	0.6251
Residuals	77.00					

Cranium Shape Lateral - MANOVA Interaction test among factors

	Df	Wilks	approx F	num Df	den Df	Pr(>F)
karn	5.00	0.0339	2.1020	125.00	265.76	2.513e-07 ***
sex	1.00	0.2288	7.1437	25.00	53.00	1.121e-09 ***
karn:sex	5.00	0.1093	1.2074	125.00	265.76	0.1040
Residuals	77.00					

LDA (percentage of correct classification) for cranium shape

Factor sex (Females: n = 52; and Males: n = 37)

<u>Cranium Shape Dorsal – LDA for "sex"</u>

Female	Male
94.23077	89.18919

<u>Cranium Shape Ventral – LDA for "sex"</u>

Female	Male
100.00000	86.48649

<u>Cranium Shape Lateral – LDA for "sex"</u>

Female	Male
100.0000	97.2973

Factor karyotypic block (A: n = 25; B: n = 17; C: n = 18; and D: n = 29)

<u>Cranium Shape Dorsal – LDA for "karyotypic block"</u>

A	B	C	D
92.00000	82.35294	83.33333	86.20690

<u>Cranium Shape Ventral – LDA for "karyotypic block"</u>

A	B	C	D
80.00000	70.58824	66.66667	86.20690

<u>Cranium Shape Lateral – LDA for "karyotypic block"</u>

A	B	C	D
76.00000	82.35294	77.77778	86.20690

Factor karyotypic number (2n = 54(A): n = 19; 2n = 2n = 54(C): n = 18; 2n = 55a: n = 6; 2n = 55b: n = 5; 2n = 56b: n = 24; 2n = 58: n = 17)

Cranium Shape Dorsal – LDA for “karyotypic number”

54(A)	54(C)	55a	55b	56b	58
73.68421	61.11111	0.00000	60.00000	70.83333	70.58824

Cranium Shape Ventral – LDA for “karyotypic number”

54(A)	54(C)	55a	55b	56b	58
68.42105	61.11111	33.33333	0.00000	54.16667	70.58824

Cranium Shape Lateral – LDA for “karyotypic number”

54(A)	54(C)	55a	55b	56b	58
68.42105	72.22222	66.66667	80.00000	91.66667	82.35294

Factor larger groups (AB: n = 42; CD: n = 47)

Cranium Shape Dorsal – LDA for “larger groups”

AB	CD
85.71429	93.61702

Cranium Shape Ventral – LDA for “larger groups”

AB	CD
88.09524	87.23404

Cranium Shape Lateral – LDA for “larger groups”

AB	CD
83.33333	80.85106

Factor chromosome pair 1 (AA [acrocentric homozygote]: n = 41; MA [heterozygote]: n = 5; MM [metacentric homozygote]: n = 43)

Cranium Shape Dorsal – LDA for “pair 1”

AA	MA	MM
78.04878	80.00000	72.09302

Cranium Shape Ventral – LDA for “pair 1”

AA	MA	MM

60.97561	0.00000	69.76744

<u>Cranium Shape Lateral – LDA for "pair 1"</u>

AA	MA	MM
80.4878	80.0000	74.4186

Factor chromosome pair 2 (AA [acrocentric homozygote]: n = 17; MA [heterozygote]: n = 6; MM [metacentric homozygote]: n = 66)

<u>Cranium Shape Dorsal – LDA for "pair 2"</u>

AA	MA	MM
64.70588	16.66667	95.45455

<u>Cranium Shape Ventral – LDA for "pair 2"</u>

AA	MA	MM
70.58824	0.00000	96.96970

<u>Cranium Shape Lateral – LDA for "pair 2"</u>

AA	MA	MM
47.05882	50.00000	93.93939
